# Au^3+^/Au^0^ Supported on Chromium(III) Terephthalate Metal Organic Framework (MIL-101) as an Efficient Heterogeneous Catalystfor Three-Component Coupling Synthesis of Propargylamines

**DOI:** 10.3390/ma10020099

**Published:** 2017-01-25

**Authors:** Lili Liu, Xishi Tai, Xiaojing Zhou

**Affiliations:** School of Chemistry & Chemical Engineering and Environmental Engineering, Weifang University, Weifang 261061, China; zhouxiaojing105@wfu.edu.cn

**Keywords:** metal-organic frameworks, MIL-101, post-synthesis modification, three-component coupling reactions, propargylamines

## Abstract

Post-synthesis modification is a useful method for the functionalization of metal–organic frameworks (MOFs). A novel catalyst Au@MIL-101-ED-SA (ED = ethylenediamine, SA = salicylaldehyde), containing coexisting Au^3+^ ions and Au^0^ nanoparticles, was prepared successfully by post-synthesis modification with ethylenediamine, salicylaldehyde and gold. Gold nanoparticles supported on MIL-101 (Au@MIL-101) were prepared successfully by the impregnation method. Au@MIL-101-ED-SA and Au@MIL-101 were characterized by N_2_ adsorption–desorption, X-ray diffraction, infrared spectroscopy, thermogravimetric analysis, X-ray photoelectron spectroscopy, and inductively coupled plasma-optical emission spectrometry. Au@MIL-101-ED-SA and Au@MIL-101 were applied as environmentally friendly catalysts in the three-component coupling reaction of aldehydes, amines, and alkynes for the preparation of diverse propargylamines. Au@MIL-101-ED-SA contained a fraction of cationic gold (Au^3+^/Au^0^ = 0.9) and showed higher catalytic activity than Au@MIL-101, which was prepared by the impregnation method. Furthermore, the reactions were performed under heterogeneous conditions and the novel catalyst was successfully recycled for four consecutive runs.

## 1. Introduction

The transition-metal-catalyzed reaction of aldehyde, alkyne, and amine (A^3^ coupling) is an important and facile method to produce propargylamines compared with traditional methods, such as the reaction of less commercially available propargyl halides with amines or using stoichiometric amounts of lithium or magnesium acetylides with imines [1,2,3,4]. The obtained propargylamines are versatile synthetic intermediates in organic synthesis and are also important structural elements in natural products and therapeutic drug molecules, and are used for the synthesis of polyfunctional amino derivatives [4,5,6]. In recent years, various transition metals such as gold [7,8], copper [1], silver [4,9], indium [10,11], iron [12], zinc [13], nickel [14] have been extensively used for the synthesis of propargylamines via the C–H alkyne activation; among which, the cationic gold species showed the highest catalytic activity [7,15]. However, besides the potential limitations of homogeneous catalysis for achieving a sustainable catalytic process, the rapid reduction of cationic gold species into inactive metallic atoms is also unavoidable when gold salts activate alkynes/alkenes [16]. Heterogeneous catalysis offers the opportunity for easy separation and recycling of the catalyst. Environmentally benign, efficient, and economical synthesis of propargylamines has become more important to address industrial and environmental concerns [4]. Translation of homogeneous catalysis into heterogeneous catalysis is a promising solution to green and sustainable development in the chemical industry. Recent research has shown that metal–organic frameworks (MOFs) could bridge the gap between homogeneous and heterogeneous catalysis [7,8,17].

MOFs have attracted considerable attention because of their enormous varieties of interesting molecular topologies and wide potential applications as functional materials [18,19,20,21,22,23,24,25,26,27,28]. In recent years, numerous studies have focused on the heterogenization of homogeneous catalysts based on MOFs [29,30,31,32,33,34]. An important characteristic of MOFs is the possibility of post-synthesis modification as a practical approach to incorporate a wide range of functional groups into MOFs to produce materials with new functionalities [35,36,37,38,39]. Post-synthesis modification could be implemented to impart functionality to MOFs depending on their intrinsic chemical and structural characteristic [40,41,42,43,44,45]. Recently, Kutzscher et al. successfully synthesized proline functionalization UiO-67 and UiO-68 catalysts (UiO-67-NHPro and UiO-68-NHPro) by post-synthesis modification [21]. Both catalysts showed reversed diastereoselectivity in aldol addition, preferring syn-aldol adduct formation for reaction of cyclohexanone with 4-nitrobenzaldehyde, in contrast to homogeneous catalysis preferring the antialdol adduct [21]. Additionally, Saedi et al. synthesized two MOF-supported Mo(VI) oxidation catalysts bearing N,O-chelating ligand via dative and covalent-dative post-synthesis modifications of a two-fold interpenetration pillared-layer amine-functionalized MOF known as TMU-16-NH_2_ [19]. Both of the modified MOFs exhibited good selectivity toward epoxide compared with the other oxidative products, and full reusability for alkenes epoxidation by *tert*-butylhydroperoxide.

Herein, we report a new gold-functionalized MIL-101 catalyst (Au@MIL-101-ED-SA) which was synthesized by post-synthesis modification. The catalyst, Au@MIL-101-ED-SA, which contains a fraction of cationic gold (Au^3+^/Au^0^ = 0.9) exhibits a higher catalytic activity in the three-component coupling reactions of aldehydes, amines, and alkynes compared with that of Au@MIL-101 prepared by the impregnation method. Furthermore, the synthesized gold-functionalized MIL-101 catalyst can be readily centrifugated and separated from the reaction solution, allowing for recycling of the catalyst four times.

## 2. Results and Discussion

### 2.1. Catalyst Synthesis and Characterization

**Scheme 1:**
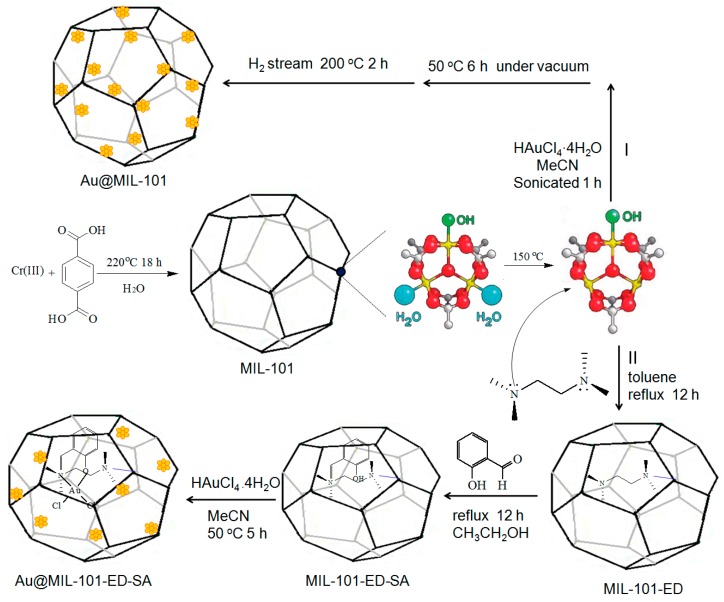
The strategy for gold-functionalized MIL-101 catalysts: (**I**) Au@MIL-101; (**II**) Au@MIL-101-ED-SA.

In our previous studies, the condensation of the amine-functionalized framework IRMOF-3 and salicylaldehyde has been reported [7]. In a parallel approximation, we have also used the post-synthesis methodology to prepare MOFs with Au^3+^/Au^0^-containing catalyst with potential use in catalysis. Chromium(III) terephthalate MIL-101 with molecular formula Cr_3_(OH)(H_2_O)_2_O[(O_2_C)C_6_H_4_(CO_2_)]_3_·*n*H_2_O (*n* ≈ 25) was selected as a parent framework for the synthesis of gold-functionalized MOF catalysts because (1) it has a large pore size (2.9 to 3.4 nm) and very large Langmuir surface area (5900 m^2^/g); (2) it has long-term stability in most organic solvents, water, and air atmospheres; (3) it has numerous potential unsaturated chromium sites (up to 3.0 mmol/g) [40,42,46]. The coordinatively unsaturated metal sites allow grafting of organic ligand [47,48]. Scheme 1 shows the strategy for gold-functionalized MIL-101 catalysts Au@MIL-101 and Au@MIL-101-ED-SA. In the present study, the MIL-101 was synthesized in deionized water, without the addition of toxic and corrosive hydrofluoric acid that is typically used in the standard preparation of MIL-101 [49]. MIL-101 was hydrothermally synthesized at 220 °C for 18 h using chromium(III) nitrate nonahydrate (Cr(NO_3_)_3_∙9H_2_O), terephthalic acid, and water [28,50,51,52]. The as-synthesized MIL-101 was further purified using hot ethanol under reflux to remove the remaining unreacted ligands trapped in the pores. Then, MIL-101 was treated at 150 °C for 12 h under vacuum to remove the terminal water molecules, thus providing coordinatively unsaturated metal sites for further functionalization. For functionalization of coordinatively unsaturated metal sites of chromium(III) of MIL-101, ethylenediamine (ED) was chosen as a grafting reagent. The ethylenediamine-grafted MIL-101, named MIL-101-ED, was synthesized by coordination of ethylenediamine to the dehydrated MIL-101, performed in toluene by heating to reflux for 12 h. As shown in Scheme 1, one amine group of ethylenediamine is linked to a coordinatively unsaturated metal site of chromium(III) of MIL-101 by direct ligation, the other amine group plays the role of an anchoring group to stabilize other organic linkers. Then, MIL-101-ED-SA was obtained by inducing the reaction of the amine groups of MIL-101-ED with salicylaldehyde to form the corresponding salicylideneimine. The final step to prepare the Au^3+^/Au^0^ containing the catalyst, Au@MIL-101-ED-SA, consisted in reacting a suitable gold precursor, HAuCl_4_, with the imine-modified material. Supported Au^0^ nanoparticles were prepared by impregnation of activated MIL-101 with a HAuCl_4_ precursor that was diluted in acetonitrile. The sample was sonicated for 1 h and aged at room temperature for 12 h. The as-synthesized catalyst was dried under vacuum at 50 °Cfor 6 h and then was treated in a stream of H_2_ at 200 °C for 2 h to yield Au@MIL-101.

**Figure 1:**
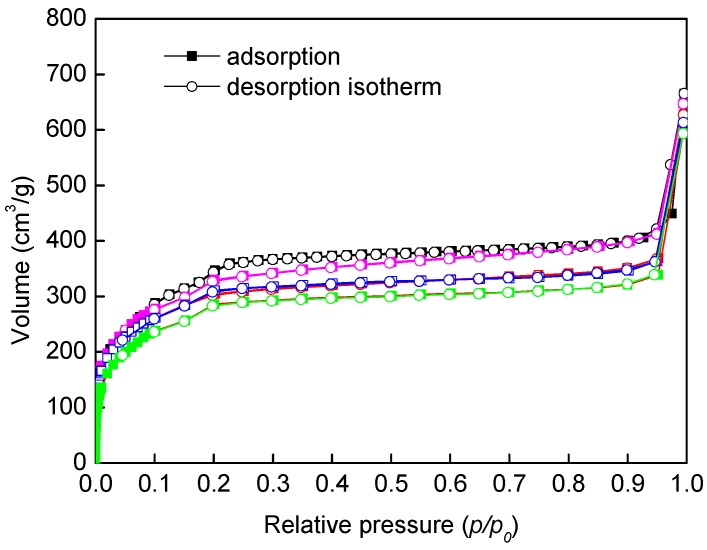
N_2_ adsorption–desorption isotherm of MIL-101 (black), MIL-101-ED (magenta), MIL-101-ED-SA (red), Au@MIL-101-ED-SA (green), and Au@MIL-101 (blue).

**Figure 2:**
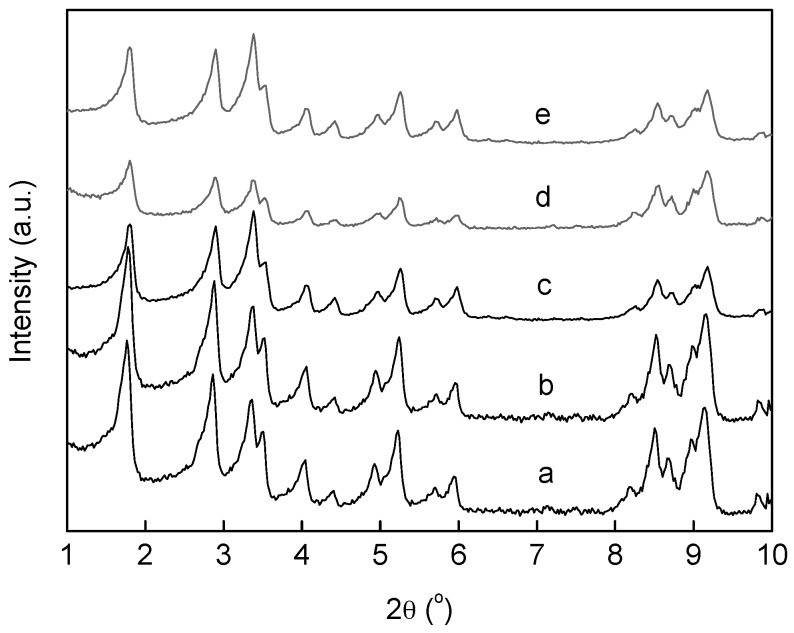
XRD patterns of the different MOFs samples: (**a**) MIL-101; (**b**) MIL-101-ED; (**c**) MIL-101-ED-SA; (**d**) Au@MIL-101-ED-SA; and (**e**) Au@MIL-101.

**Figure 3:**
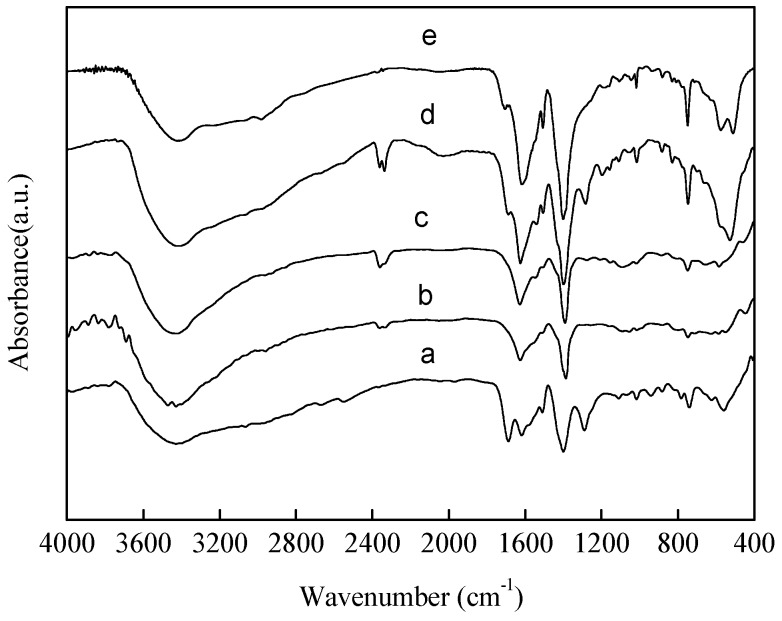
Infrared spectra of MOF samples: (**a**) MIL-101; (**b**) MIL-101-ED; (**c**) MIL-101-ED-SA; (**d**) Au@MIL-101-ED-SA; and (**e**) Au@MIL-101.

**Figure 4:**
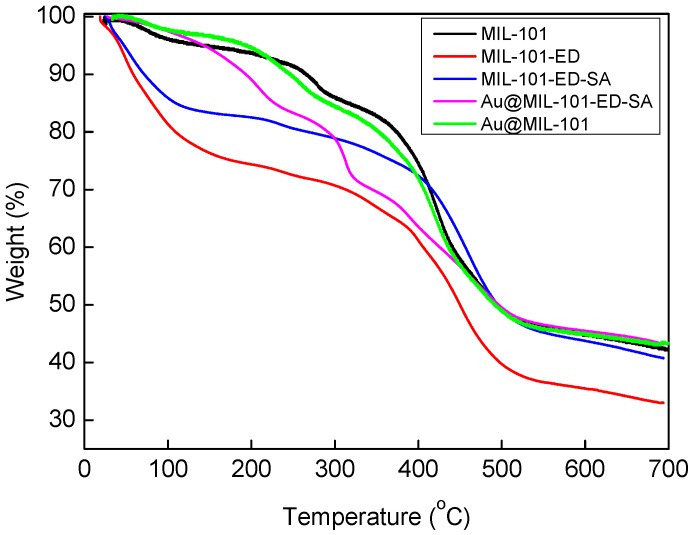
TG curves of different MOF samples.

**Figure 5:**
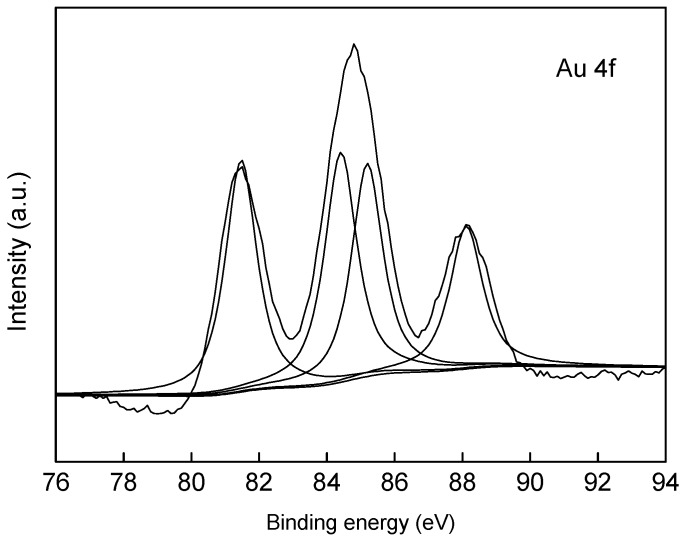
XPS spectra of Au 4f in Au@MIL-101-ED-SA.

The samples were characterized by various techniques including nitrogen adsorption–desorption measurements, powder X-ray diffraction (PXRD), IR spectroscopy, thermogravimetric analysis, and X-ray photoelectron spectroscopy (XPS). The nitrogen adsorption-desorption isotherms of activated MIL-101 and postmodified MIL-101 exhibited characteristics of type IV isotherms (Figure 1). The BET surface area of MIL-101 is 1186 m^2^/g. The pore volume and average pore size calculated by the Barrett-Joyer-Halenda model from the nitrogen desorption isotherm are 0.49 cm^3^/g and 2.8 nm. The BET surface area value is lower than that reported in the literature [49,53]. Higher BET surface areas could be obtained by using HF rather than H_2_O as solvent [49,53]. The BET surface area of MIL-101 was highly affected by the synthetic raw material. Férey synthesized MIL-101 with a BET surface area of 5900 m^2^/g consists in the hydrothermal reaction of terephthalic acid, with Cr(NO_3_)_3_∙9H_2_O, fluorhydric acid, and H_2_O for 8 h at 220 °C [49]. Bromberg and Hatton synthesized MIL-101 hydrothermally, utilizing autoclave oven heat supply using terephthalic acid, Cr(NO_3_)_3_∙9H_2_O, and deionized water with a BET surface area of 3460 m^2^/g [53]. Moreover, our procedure for synthesizing MIL-101 was not under N_2_ protection, although MOFs adsorb moisture very quickly upon exposure to ambient air [7,54,55]. In addition, the organic linkers and the solvent molecules such as H_2_O could be not completely removed before BET measurement. The nitrogen sorption study revealed that the BET surface area of MIL-101-ED, MIL-101-ED-SA, Au@MIL-101-ED-SA, and Au@MIL-101 was 1096 m^2^/g, 1004 m^2^/g, 942 m^2^/g, and 1020 m^2^/g, respectively. The experimental result shows a decrease in BET after MIL-101 was modified due to the added ethylenediamine, salicylaldehyde, and gold to MIL-101. Similar results have been observed in other MOFs after postmodification [56,57]. The X-ray diffraction (XRD) pattern of MIL-101 is very similar to those reported in the literature (Figure 2a) [49]. The post-synthesis modification with ethylenediamine and salicylaldehyde did not result in any apparent loss of crystallinity in the X-ray diffraction patterns, indicating that the integrity of the MIL-101 framework was maintained (Figure 2b,c). Both Au@MIL-101-ED-SA and Au@MIL-101 have XRD patterns similar to that of MIL-101 supporting the retention of the parent framework structure (Figure 2c,d). However, IRMOF-3 modified by salicylaldehydeand gold with the post-synthesis method affected the variance of the intensity ratio for the typical two peaks [7]. The change in XRD pattern could be due to the deviation or shrinkage, and even the collapse of pores during the modification process. Figure 3 shows the IR spectra of the bare MIL-101 compound compared with four samples after ethylenediamine, salicylaldehyde, and gold grafting. The IR spectrum of MIL-101 exhibiting an absorption band at around 1700 cm^−1^ can be attributed to free terephthalic acid ligands that are encapsulated within the pores of the structure in their protonated form (–CO_2_H) [8,55,58,59]. IR spectroscopy showed N–H stretching bands at 3477 and 3428 cm^−1^ for MIL-101-ED, supporting the successful incorporation of the molecular of ethylenediamine in the postmodified MIL-101. The N–H stretches at 3477 and 3428 cm^−1^, associated with the amine of the ethylenediamine, are notably diminished for MIL-101-ED-SA and Au@MIL-101-ED-SA samples, which indicated the formation of salicylideneimine by reaction of the amine of the ethylenediamine with the aldehyde group of the salicylaldehyde [7,60]. The TG curves of all samples show three steps of weight loss stages in the temperature range from 25 to 700 °C (Figure 4). The first stage ranging from 25 to 100 °C was due to the release of physically adsorbed solvent molecules such as ethanol, toluene, and acetonitrile. The second stage from 100 to 300 °C was ascribed to the release of unreacted terephthalic acid, ethylenediamine, and salicylaldehyde. The third stage of weight loss over temperatures higher than 300 °C was due to the organic decomposition. The XPS investigation of Au@MIL-101-ED-SA at the gold 4f level indicated that Au@MIL-101-ED-SA contains a fraction of cationic gold, and the ratio of Au^3+^/Au^0^ for Au@MIL-101-ED-SA is 0.9 (Au^3+^/Au^0^ = 0.9) (Figure 5). This value is higher than that 4.6%Au/IRMOF-3 (Au^3+^/Au^0^ = 0.2). ICP-OES analysis indicated that the chromium and gold weight loading were 7.65 wt % and 4.89 wt % in Au@MIL-101-ED-SA, and the Cr:Au ratio appears to be 6:1, corresponding to 16.7% of the Cr sites had been functionalized. The chromium and gold contents of Au@MIL-101 were 8.42 wt % and 4.89 wt % as determined by ICP-OES, and the Cr:Au ratio is 6.5:1.

### 2.2. Catalytic Studies

**Figure 6:**
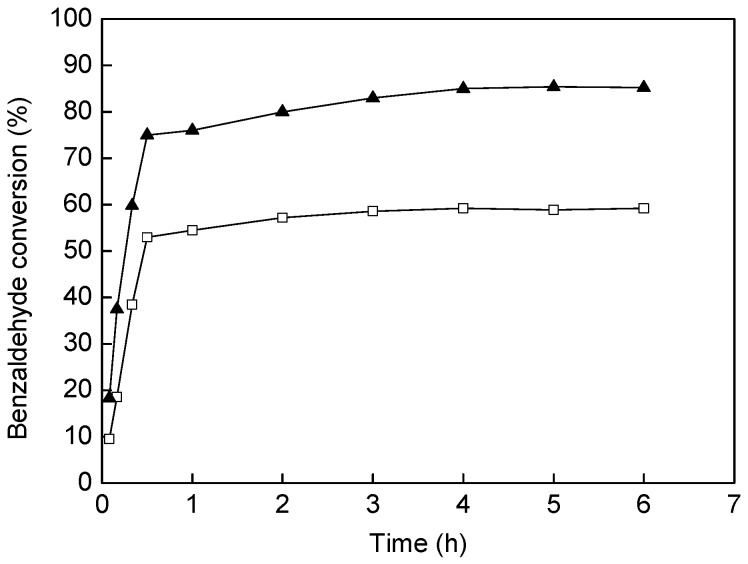
Comparison of the catalytic activity of Au@MIL-101-ED-SA (▲) and Au@MIL-101 (□) catalysts under identical reaction conditions (benzaldehyde (0.25 mmol), piperidine (0.30 mmol), and phenylacetylene (0.33 mmol), 1,4-dioxane (1.5 mL), catalyst (0.06 g), 120 °C).

The catalytic activity of Au@MIL-101-ED-SA and Au@MIL-101 was tested for the A^3^ coupling reaction of aldehyde, amine, and alkyne. Benzaldehyde, piperidine, and phenylacetylene with 1,4-dioxane as solvent were used as model substrates to study the catalytic activity of the different supported gold catalysts. Initially, the effect of temperature was studied to optimize the reaction condition. At higher temperatures, Au@MIL-101-ED-SA exhibits good catalytic activity, at 120 °C for 4 h, 85% conversion of benzaldehyde was obtained, however lower conversions are obtained at 80 °C and 100 °C. The conversions of benzaldehyde were 18.5% and 43.6% over Au@MIL-101-ED-SA at 80 °C and 100 °C for 4 h. Figure 6 shows the kinetic curves for Au@MIL-101-ED-SA and Au@MIL-101 for the A^3^ coupling reaction of benzaldehyde, phenylacetylene, and piperidine with 1,4-dioxane as solvent at 120 °C. Au@MIL-101-ED-SA has a higher conversion of benzaldehyde than Au@MIL-101. Maximum conversions of 85.0% and 59.0% were obtained within 4 h over Au@MIL-101-ED-SA and Au@MIL-101, and then remained constant. Moreover, the catalysts feature a selectivity of 100% to the product of propargylamine for the A^3^ coupling reaction. The reaction rates from the initial rates from Figure 6 are calculated to be 187.1 and 95.8 mmolg_Au_^−1^·h^−1^ for Au@MIL-101-ED-SA and Au@MIL-101 based on the total gold content of the catalyst. If one calculates the turnover frequency (TOF) based on the initial conversion of benzaldehyde taking into account the total gold content of the catalyst, the values obtained are 36.9 h^−1^ and 18.9 h^−1^ for Au@MIL-101-ED-SA and Au@MIL-101, respectively. The catalyst of Au@MIL-101-ED-SA, prepared by post-synthesis modification, contains a fraction of cationic gold, which gives a higher TOF than that of Au@MIL-101 prepared by the impregnation method. In our previous work, a conversion of 77% was obtained within 0.5 h over 4.6%Au/IRMOF-3 [7]. The reaction rates and TOF values obtained are 120.5 mmolg_Au_^−1^·h^−1^ and 23.7 h^−1^, respectively, for 4.6%Au/IRMOF-3 taking into account the total gold content of the catalyst. Compared with 4.6%Au/IRMOF-3, Au@MIL-101-ED-SA gives a higher reaction rates and TOF. According to the previous report [8,17], the activity of Au-based catalysts for the A^3^ coupling reaction decreases in the following order: Au^3+^ > Au^0^. Thus, it is not surprising that the TOF of Au@MIL-101-ED-SA is larger than that 4.6%Au/IRMOF-3 since it has been found that in the latter catalyst, the fraction of Au^3+^ with respect to total gold is only 0.2.

To examine the scope and the generality of this three-component coupling reaction on the gold-functionalized catalysts of Au@MIL-101-ED-SA and Au@MIL-101, we extended our studies to different combinations of aldehydes, amines, and alkynes. The results are summarized in Table 1. The Au@MIL-101-ED-SA and Au@MIL-101 are general catalysts for the three-component-coupling, as can be deduced by reacting various aldehydes, amines, and alkynes. Both aromatic and aliphatic aldehydes were able to undergo the corresponding three-component-coupling, and afforded good conversions of aldehyde in the A^3^ coupling reaction with Au@MIL-101-ED-SA and Au@MIL-101 at 120 °C for 4 h (Table 1, entries 1–7, 16–22). Aryl aldehydes possessing electron-withdrawing groups afforded a higher conversion of aldehydes than aryl aldehydes with the electron-donating group over Au@MIL-101-ED-SA and Au@MIL-101 (Table 1, entries 2–4, 17–19). The coupling of aliphatic aldehydes cyclohexanecarboxaldehyde, *n*-heptylaldehyde, and *n*-octaldehyde led to propargylamines in 92.9%, 98.8%, and 97.6% conversions with the catalyst of Au@MIL-101-ED-SA, respectively (Table 1, entries 5–7). Au@MIL-101 was also found to have a good activity for aliphatic aldehyde, leading to 93.1%, 98.4%, and 98.3% conversion of cyclohexanecarboxaldehyde, *n*-heptylaldehyde, and *n*-octaldehyde, respectively (Table 1, entries 20–22). Alicyclic amines such as pyrrolidine and morpholine gave moderate conversions, whereas diethylamine afforded a lower conversion over Au@MIL-101-ED-SA and Au@MIL-101 (Table 1, entries 8–10 and 23–25). This was probably due to the iminium ions generated from alicyclic amines and benzaldehyde which are more stable than that generated from dialkyl amine and benzaldehyde. The Au@MIL-101-ED-SA and Au@MIL-101 catalyzed three-component coupling reaction also worked well for various alkynes except 1-octyne. It was not affected by the steric hindrance of the alkynes. When increasing chain length, it gave a similar conversion of benzaldehyde. The conversions were 52.8%, 51.3%, and 51.7% for methyl-, ethyl-, and butyl-substituted phenylacetylene with Au@MIL-101-ED-SA, respectively. Furthermore, the benzaldehyde conversions were 31.5%, 32.9%, and 33.9% for methyl-, ethyl-, and butyl-substituted phenylacetylene with Au@MIL-101, respectively. The reaction worked well for (trimethylsilyl)acetyleneand benzaldehyde conversions are 90.9% and 52.6% for Au@MIL-101-ED-SA and Au@MIL-101, respectively. Only a trace amount of product was obtained with 1-octyne over Au@MIL-101-ED-SA and Au@MIL-101.

To check the reusability, Au@MIL-101-ED-SA and Au@MIL-101 were repeatedly filtered out and subjected to a new reaction batch without any further treatment. Table 2 summarizes the benzaldehyde conversion in four consecutive cycles on the catalysts of Au@MIL-101-ED-SA and Au@MIL-101. It has a sharp decrease of the catalytic activity in the second reaction cycle over Au@MIL-101-ED-SA. However, a slight decrease of the catalytic activity was found in the third and fourth reaction cycles. Significant differences are observed with the catalyst of Au@MIL-101. Au@MIL-101 catalysts show only a small decrease in the benzaldehyde conversion in the second, third and fourth reaction cycles. We analyzed the gold content after four reaction cycles to check if the activity loss of the catalyst is due to gold leaching in the reaction medium. As evidenced by inductively coupled plasma-optical emission spectrometry (ICP–OES) analysis, ca. 5 wt % leaching of gold was found over the recycled Au@MIL-101-ED-SA. Therefore, the activity loss of Au@MIL-101-ED-SA in the second catalytic cycle could be due to the leaching of gold. The Au^3+^ species are easily reduced even at room temperature, and alkynes, alkenes alcohols, CO, and phosphines in the reaction media are the reducing agents [7,61]. The sharp decrease of catalytic activity of Au@MIL-101-ED-SA in the second cycle could be also explained by the decrease of Au^3+^ sites.

## 3. Experimental Section

### 3.1. General Information

All chemicals were obtained from Sigma–Aldrich (Milwaukee, WI, USA) and were used without further purification. ICP emission spectrometry (Perkin–Elmer Optima 7000DV ICP-OES spectrometer, PerkinElmer, Waltham, MA, USA) was used for the analysis of the chromium and gold content of the synthesized supported gold catalysts. The crystalline materials were analyzed by XRD using a Brüker D8 with CuKα radiation (Karlsruhe, Germany). Thermogravimetric analysis of the samples was performed by means of a TAQ600, under flowing N_2_ at heating rates of 10 K/min. Nitrogen adsorption at 77 K in a Quantachrome Autosorb-1 gas adsorption analyzer (Boynton Beach, FL, USA) was used to determine the textural properties of the MOFs. Infrared (IR) spectra (400–4000 cm^−1^) were recorded from KBr pellets in a Brüker Tensor 27 spectrometer (Karlsruhe, Germany). XPS measurements were carried out in a Thermo ESCALAB 250XI instrument (Waltham, MA, USA).

### 3.2. Catalyst Synthesis

#### 3.2.1. Synthesis of MIL-101

Chromium(III) nitrate nonahydrate (Cr(NO_3_)_3_∙9H_2_O, 1.0 g, 2.5 mmol) and terephthalic acid (0.42 g, 2.5 mmol) were dissolved in deionized water (10 mL) and sonicated for 1 h at room temperature in air, resulting in a dark blue-colored suspension. The suspension was placed in a 100 mL Teflon-lined steel autoclave and kept in an oven at 220 °C for 18 h without stirring. After the synthesis and equilibration at room temperature, the green powders were separated from water using a centrifuge (10,000 r/min, 6 min). A significant amount of nonreacted terephthalic acid is present both outside and within the pores of MIL-101 [40,49]. To avoid this, the as-synthesized MIL-101 was activated in boiling ethanol under reflux for 24 h to remove the remaining unreacted ligands trapped in the pores. The products were separated by centrifugation and dried at 150 °C for 12 h under vacuum [29,50,51,52].

#### 3.2.2. Synthesis of EthylenediamineFunctionalized MIL-101

The ethylenediamine functionalized MIL-101 was prepared according to the following process. In a typical procedure, MIL-101 (0.4 g) was suspended in 15 mL toluene. To this suspension, an appropriate amount (0.6 mmol, 36.0 mg) of ethylenediamine was added and the mixture was stirred under reflux for 12 h. The product was recovered by centrifugation and washed with ethanol. The products were dried at 50 °C for 5 h under vacuum. MIL-101-ED denotes the MIL-101 grafted with ethylenediamine [40].

#### 3.2.3. Synthesis of SalicylaldehydeFunctionalized MIL-101

The MIL-101-ED (0.76 g) was dispersed in 6 mL ethanol. To this slurry, a solution of salicylaldehyde (1.1 g) in ethanol (6 mL) was dropwise added at room temperature. The mixture was stirred under reflux for 12 h. Then, the sample was collected by centrifugation and dried under vacuum at 50 °C for 5 h.

#### 3.2.4. Synthesis of Au@MIL-101-ED-SA

A solution of HAuCl_4_∙4H_2_O (0.053 g) in MeCN (0.8 mL) was dropwise added to the MIL-101-ED-SA (0.50 g) at room temperature. The sample was sonicated at room temperature for 1 h. Then, the as-synthesized sample was aged at room temperature for 12 h and dried under vacuum at 50 °C for 5 h to yield Au@MIL-101-ED-SA [7,17]. The gold and chromium contents of Au@MIL-101-ED-SA were 4.89 wt % and 7.65 wt %, as determined by ICP-OES.

#### 3.2.5. Synthesis of Au@MIL-101

Au@MIL-101 was prepared by an impregnation method. In a typical synthesis, a solution of HAuCl_4_∙4H_2_O (0.053 g) in 0.8 mL MeCN was dropwise added to MIL-101 (0.5 g) at room temperature and was sonicated for approximately 1 h. Then, the sample was stored at room temperature for 12 h. The sample was dried at 50 °C for 6 h under vacuum, followed by treating in a stream of H_2_ (50 mL/min) at 200 °C for 2 h to yield Au@MIL-101 [41,50,55]. The resulting gold and chromium loading were 4.96 wt % and 8.42 wt % as determined by ICP-OES.

### 3.3. General Procedure for the Three-Component Coupling Reaction

In a typical experiment of the three-component (A^3^) coupling reaction, three reactants such as benzaldehyde (0.25 mmol), piperidine (0.30 mmol), and phenylacetylene (0.33 mmol) were mixed in 1.5 g of 1,4-dioxane as a solvent and then 60 mg of the dried catalyst was rapidly added into the reactor. The reactor was placed in a 120 °C oil bath. At the end of each catalytic A^3^ coupling reaction, the catalyst was isolated by centrifugation from the reaction solution, dried at 150 °C under vacuum, and then reused in the second run of each reaction. The products were analyzed using a gas chromatograph (GC-1100) equipped with a SE-54 capillary column and a hydrogen flame detector. The conversions were calculated based on *n*-nonane as an internal standard.

## 4. Conclusions

Post-synthesis modification is a useful method for the functionalization of MOFs. New heterogeneous gold-functionalized catalysts containing a fraction of cationic gold (Au^3+^/Au^0^ = 0.9) have been synthesized by the post-synthesis modification strategy using the metal–organic framework, MIL-101. The structure of gold-functionalized catalyst was well retained after post-synthesis modification. The newly developed gold-functionalized MIL-101 catalyst, prepared by the post-synthesis modification approach, exhibited higher catalytic activity in the three-component coupling reaction than Au@MIL-101 synthesized by the impregnation method. This work demonstrates the great versatility of MOFs as platforms for the design of new catalysts and offers a better understanding of gold-functionalized catalysts.

## Figures and Tables

**Table 1:** **The** A^3^ coupling reaction of different aldehydes, amines and alkynes catalyzed by Au@MIL-101-ED-SA and Au@MIL-101 ^1^.

Entry	Catalyst	R_1_	R_2_R_3_NH	R_4_	T/h	Conv./%
1	Au@MIL-101-ED-SA	Ph	piperidine	Ph	0.5	75.0
2	Au@MIL-101-ED-SA	3-ClC_6_H_4_	piperidine	Ph	4	95.2
3	Au@MIL-101-ED-SA	4-MeC_6_H_4_	piperidine	Ph	4	23.1
4	Au@MIL-101-ED-SA	4-MeOC_6_H_4_	piperidine	Ph	4	79.1
5	Au@MIL-101-ED-SA	Cyclohexyl	piperidine	Ph	4	92.9
6	Au@MIL-101-ED-SA	Heptyl	piperidine	Ph	4	98.8
7	Au@MIL-101-ED-SA	Hexyl	piperidine	Ph	4	97.6
8	Au@MIL-101-ED-SA	Ph	pyrrolidine	Ph	4	57.3
9	Au@MIL-101-ED-SA	Ph	morpholine	Ph	4	45.0
10	Au@MIL-101-ED-SA	Ph	diethylamine	Ph	4	8.8
11	Au@MIL-101-ED-SA	Ph	piperidine	4-MeC_6_H_4_	4	52.8
12	Au@MIL-101-ED-SA	Ph	piperidine	4-EtC_6_H_4_	4	51.3
13	Au@MIL-101-ED-SA	Ph	piperidine	4-ButC_6_H_4_	4	51.7
14	Au@MIL-101-ED-SA	Ph	piperidine	Hexyl	4	12.9
15	Au@MIL-101-ED-SA	Ph	piperidine	(CH_3_)_3_Si	4	90.9
16	Au@MIL-101	Ph	piperidine	Ph	0.5	53.0
17	Au@MIL-101	4-MeC_6_H_4_	piperidine	Ph	4	5.7
18	Au@MIL-101	4-MeOC_6_H_4_	piperidine	Ph	4	48%
19	Au@MIL-101	3-ClC_6_H_4_	piperidine	Ph	4	56.0
20	Au@MIL-101	Cyclohexyl	piperidine	Ph	4	93.1
21	Au@MIL-101	Heptyl	piperidine	Ph	4	98.4
22	Au@MIL-101	Hexyl	piperidine	Ph	4	98.3
23	Au@MIL-101	Ph	pyrrolidine	Ph	4	40.4
24	Au@MIL-101	Ph	morpholine	Ph	4	35.0
25	Au@MIL-101	Ph	diethylamine	Ph	4	6.2
26	Au@MIL-101	Ph	piperidine	4-MeC_6_H_4_	4	31.5
27	Au@MIL-101	Ph	piperidine	4-EtC_6_H_4_	4	32.9
28	Au@MIL-101	Ph	piperidine	4-ButC_6_H_4_	4	33.9
29	Au@MIL-101	Ph	piperidine	Hexyl	4	7.3
30	Au@MIL-101	Ph	piperidine	(CH_3_)_3_Si	4	52.6

Note: ^1^ 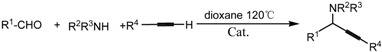, Reaction conditions: aldehyde (0.25 mmol), amine (0.30 mmol), alkyne (0.33 mmol), catalyst (0.06 g).

**Table 2:** Recyclability of Au@MIL-101-ED-SA and Au@MIL-101 in the A^3^ coupling reaction of benzaldehyde, piperidine, and phenylacetylene ^1^.

Run	Catalyst	Time/h	Yield/%	Catalyst	Time/h	Yield/%
1	Au@MIL-101-ED-SA	0.5	75	Au@MIL-101	0.5	53.0
2	Au@MIL-101-ED-SA	4	50.0	Au@MIL-101	4	52.3
3	Au@MIL-101-ED-SA	4	45.5	Au@MIL-101	4	48.6
4	Au@MIL-101-ED-SA	4	43.8	Au@MIL-101	4	47.8

Note: ^1^ Reaction conditions: benzaldehyde (0.25 mmol), piperidine (0.30 mmol), phenylacetylene (0.33 mmol), and dioxane (1.5 g), catalyst (0.06 g), 120 °C.
